# Protocol for developing a surgically operated model to mimic human glioblastoma resection and recurrence

**DOI:** 10.1016/j.xpro.2026.104503

**Published:** 2026-04-17

**Authors:** Perrine Schneller, Julien Pierson, Joël Daouk, Fabien Rech, Matthieu Doyen, Sophie Pinel, Muriel Barberi-Heyob

**Affiliations:** 1 CRAN, UMR 7039, Université de Lorraine, CNRS, 54505 Vandœuvre-lès-Nancy, France; 2 Faculté de Médecine de Nancy, Université de Lorraine, 9 avenue de la Forêt de Haye, 54505 Vandœuvre-lès-Nancy, France; 3 Department of Neurosurgery, CHRU-Nancy, 54000 Nancy, France; 4 Université de Lorraine, IADI, INSERM U1254, 54000 Nancy, France

**Keywords:** Cancer, Clinical Protocol, Biotechnology and bioengineering

## Abstract

We present a protocol to develop a syngeneic surgical glioblastoma model using F98 cells in Fischer F344 rats. This model recapitulates tumor growth, surgical resection, and recurrence observed in patients. We describe procedures for F98 cell preparation, stereotactic implantation, MRI-guided tumor resection, and longitudinal MRI monitoring. This protocol provides a clinically relevant platform to investigate post-resection tumor recurrence and evaluate therapeutic strategies targeting residual disease.

## Highlights

• Instructions for orthotopic implantation of syngeneic F98 GBM cells in Fischer F344 rats
• Steps for surgical resection mimicking clinical GBM debulking procedures
• Procedures for weekly MRI monitoring of tumor growth, surgical success, and recurrence
• Guide for immunohistochemical characterization of the F98 GBM model in Fischer F344 rats

## Abstract (editor highlights)

Publisher’s note: Undertaking any experimental protocol requires adherence to local institutional guidelines for laboratory safety and ethics.

## Graphical abstract

**Figure:**
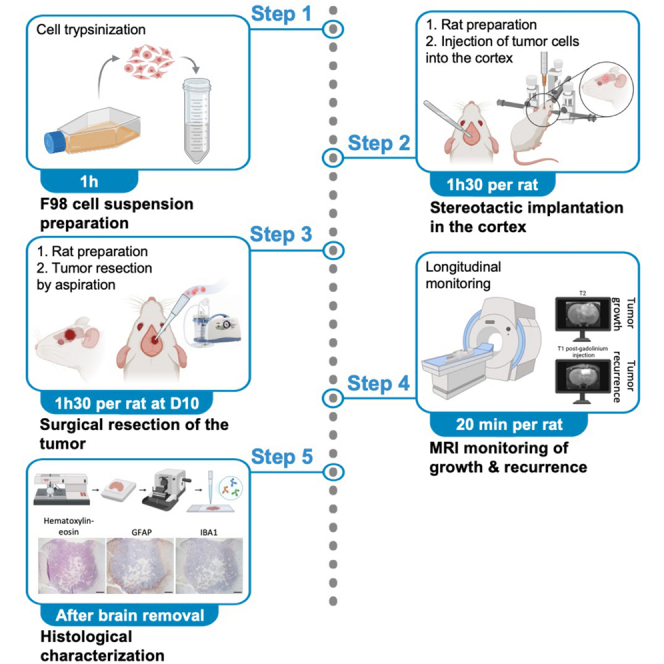

## Before you begin

Glioblastoma multiforme (GBM) is the most common and aggressive brain tumor of the central nervous system and is classified as a grade IV tumor by the World Health Organization.^1^ The standard of care, known as the Stupp protocol, combines maximal safe resection^2^ with concomitant radiotherapy and temozolomide.^3^ However, despite this multimodal approach, prognosis remains dismal with a median survival of approximately 15 months and nearly universal recurrence.

To investigate mechanisms underlying local relapse and to evaluate post-surgical therapeutic strategies, we developed a reproducible preclinical model using the F98 glioma cell line implanted orthotopically into immunocompetent Fischer F344 rats. The Fischer F344 rat strain was selected because F98 glioma cells are syngeneic to this genetic background, allowing tumor development in an immunocompetent host while preserving tumor-immune interactions. This avoids the need for immunosuppression required in xenograft models and enhances reproducibility across laboratories. The Fischer/F98 model is one of the best-characterized syngeneic GBM models in the literature and provides stable and predictable growth kinetics. The F98 model is particularly relevant due to its syngeneic nature, intrinsic resistance to ionizing radiation and chemotherapy, and its highly infiltrative growth pattern, which closely mimics the diffuse invasion seen in human GBM.^4^ Histological analyses confirm perivascular and perineuronal spread of tumor cells beyond the visible lesion, making this model well suited to study the limits of surgical resection and recurrence dynamics.

These infiltrative characteristics necessitate precise longitudinal imaging to accurately define tumor boundaries and determine the optimal timing for surgical intervention. T1-and T2-weighted MRI confirm tumor engraftment and allow non-invasive monitoring of growth, resection completeness, and recurrence. Following imaging-based validation, tumor resection is performed by surgical aspiration under stereotactic guidance, mimicking clinical neurosurgical procedures. Post-resection recurrence is tracked via MRI and confirmed histologically.

Previous studies have demonstrated that syngeneic intracranial GBM models in immunocompetent rodents recapitulate several key histopathological features of human GBM, including neovascularization, necrosis, immune-cell infiltration, and invasive growth patterns. These characteristics support the translational relevance of immunocompetent models for evaluating therapeutic strategies in a biologically complex tumor microenvironment.^5^

Beyond their histopathological relevance, several preclinical models incorporating surgical tumor resection have been developed to better recapitulate the clinical course of glioblastoma. For example, Sweeney et al. established an imageable GBM resection model using U87 xenografts combined with bioluminescence imaging and a cranial window repair technique.^6^ While this approach demonstrated the feasibility of serial imaging and survival benefit following resection, it relied on human xenografts implanted in immunodeficient mice and required genetically modified luciferase-expressing tumor cells. In contrast, the present protocol employs a syngeneic F98/Fischer F344 model in immunocompetent rats and integrates high-resolution MRI monitoring, thereby preserving tumor–immune interactions and enhancing translational relevance. Although several rodent GBM models have been previously described, detailed step-by-step protocols integrating orthotopic implantation, MRI-guided longitudinal monitoring, and standardized microsurgical resection remain scarce. In many reports, tumor induction and resection procedures are only briefly described, limiting reproducibility across laboratories. The present protocol provides a fully standardized surgical workflow, including stereotactic alignment criteria, MRI-based timing of resection, and post-operative monitoring, thereby addressing the current lack of harmonized methods for studying post-resection GBM recurrence. This model provides a robust platform for testing the efficacy of new therapies targeting residual infiltrative tumor cells in a clinically relevant context.

### Institutional permissions

The present model was developed using live vertebrate animals. All procedures were conducted in full accordance with the European Directive 2010/63/EU on the protection of animals used for scientific purposes, and adhered to the 3Rs principles (Replacement, Reduction, and Refinement) for animal welfare. Experiments were performed by qualified and authorized personnel within a certified facility (Animalerie du Campus Biologie Santé, ACBS; establishment number C 54-547-30C).

The experimental protocol was reviewed and approved by the local ethical committee (Comité d’Éthique Lorrain en Matière d’Expérimentation Animale, CELMEA; French Ethical Committee No. 66) and received authorization from the French Ministry of Research (APAFiS approval number #43911).

Seven-week-old male Fischer F344 rats (F344/HanZtmRj), weighing between 200–250 g, were obtained from Janvier Labs (Le Genest-Saint-Isle, France). Animals were housed under standard laboratory conditions: temperature 24 ± 1°C, relative humidity 50 ± 10%, in individually ventilated cages with filter tops. A reversed 12 h light/dark cycle was used to perform all procedures during the animals’ active (dark) phase. Food and water were provided ad libitum.

### F98 glioma cell maintenance in culture

**Timing: Every 7 days**

***Note:*** This section outlines the weekly subculture of F98 glioma cells.

Following initial thawing, F98 cells are maintained under standard conditions and subcultured once per week. Cells are seeded in T75 cm^2^ flasks at a density of 10^4^ cells/mL in Dulbecco’s Modified Eagle Medium (DMEM) supplemented with 10% fetal bovine serum (FBS), 1% penicillin-streptomycin, and 2% L-glutamine (store at 4°C under sterile conditions for up to 3 months). Under these conditions, F98 cells typically reach full confluence (∼100%) within 7 days.

Weekly subculturing procedure.

1. Examine cell morphology and confluence using a phase-contrast light microscope.

***Note:*** Proceed only if the monolayer is healthy and ≥90% confluent.

2. Aspirate the spent culture medium from the T75 cm^2^ flask.
3. Rinse the adherent cell layer with 3 mL of Dulbecco’s Phosphate-Buffered Saline (DPBS) to remove residual serum and debris.
4. Aspirate and discard the DPBS.
5. Add 3 mL of trypsin-EDTA solution to detach the cells.
6. Incubate the flask for 5 min at 37°C in a humidified incubator with 5% CO_2_.
7. Add 4 mL of supplemented DMEM. Pipette gently to dissociate the cells.
8. Transfer the cell suspension to a conical tube.
9. Centrifuge at 300 × *g* for 10 min at room temperature.
10. Carefully aspirate the supernatant.
11. Resuspend the cell pellet in 5 mL of fresh supplemented DMEM.
12. Determine cell concentration using a hemocytometer or automated cell counter.
13. Reseed the cells at a density of 10^4^ cells/mL in new T75 cm^2^ flasks for continued culture.

## Key resources table

**Table:** 

REAGENT or RESOURCE	SOURCE	IDENTIFIER
**Antibodies**		

GFAP	Abcam	#ab207165
IBA1	Abcam	#⁠ab178846
Goat Anti-Rabbit IgG H&L (HRP)	Abcam	#⁠ab205718

**Chemicals, peptides, and recombinant proteins**		

DMEM, high glucose (4.5 g/L), glutamine-free, phenol red-free	Thermo Fisher Scientific	Cat#31053028
Fetal Bovine Serum	Sigma-Aldrich	Cat#7524
L-Glutamine solution	Sigma-Aldrich	Cat#G7513
Penicillin-Streptomycin	Sigma-Aldrich	Cat#P0781
Sodium Pyruvate	Thermo Fisher Scientific	Cat#11360-039
Dulbecco’s Phosphate Buffered Saline (DPBS)	Sigma-Aldrich	Cat#D8537
Hanks’ Balanced Salt Solution (HBSS)	Sigma-Aldrich	Cat#H8264
Trypsin-EDTA solution	Sigma-Aldrich	Cat#T3924
Isoflurane 100%	Centravet	Cat#ISO007
Vetedine solution, 120mL	Centravet	Cat#VET001
Buprecare, 0.3mg/mL, 5x1mL	Centravet	Cat#BUP001
Ocrygel, 10 g	Centravet	Cat#OCR002
Exagon®, 100mL	Centravet	Cat#EXA001
Aqueous chloriseptin 0.05%, 60x5mL	Centravet	Cat#CHL042
Alcoholic chloriseptin 0.5%, 2x500mL	Centravet	Cat#CHL043
Lurocaïne, injectable solution, 100mL	Centravet	Cat#LUR003
Histoacryl blue sterile tissue adhesive	Centravet	Cat#COL405
DOTAREM®	Guerbet	Gadoterate Meglumine
100% ethanol	Avantor Sciences	#20821.365
96% ethanol	Avantor Sciences	#20824.365
Xylene	Avantor Sciences	#28975.325
Toluene	Avantor Sciences	#28684.364
Harris Hematoxylin	Avantor Sciences	#10047007
Erythrosin B (C. I. 45430)	Sigma-Aldrich	#1.15936.0025
Trizma® hydrochloride solution 1 M	Sigma-Aldrich	#T3038
ImmPACT NovaRED® Substrate Kit, Peroxidase (HRP)	Vector Laboratories	#SK-4805
EUKITT®	Dutscher	#124103
TWEEN® 20	Sigma-Aldrich	#P1379
Hydrogen peroxide 30%	Avantor Sciences	#1.07209.0250
Dako REAL Antibody Diluent	Agilent	#S2022
EDTA (ethylenediaminetetraacetic acid disodium salt dihydrate)	Sigma-Aldrich	#E5134
Acetic acid glacial	Avantor Sciences	#20104.298
Hydrochloric acid 37%	Avantor Sciences	#20252.290
Sigma 7-9® (Base Trizma®)	Sigma-Aldrich	#T1378

**Experimental models: Cell lines**		

F98	ATCC	CRL-2397

**Experimental models: Organisms/strains**		

6-week-old male Fischer F344 rats	Janvier Laboratories	F344/Rj Rat albinos -*Tyr*^*c*^*/Tyr*^*c*^ 6-week-old male rats

**Software and algorithms**		

GraphPad Prism	https://www.graphpad.com/	RRID: SCR_002798

**Other**		

Model 963 Ultra Precise Small Animal Stereotaxic	PhymepKopf Instruments	Cat#963
1701 RN 10UL SYR W/O NEEDLE	Phymep	Cat#7653-01
Surgical aspirator New Askir 30, 1 L container, 30L/min	Centravet	Cat#ASP712
Brushless High Speed Rotary Handpiece	Foredom	Cat#H.MH-150
Neuro Patch 2x10cm	Centravet	Cat#SPEMATE
TachoSil® Fibrin Sealant Patch	Corza Medical	N/A
Scalpel n°15, sterile box	Centravet	Cat#BIS018
Suture thread Prolene 5.0	Centravet	Cat#PR0102
Osalia 25G orange hypodermic needle	Centravet	Cat#AIG484
Guthrie Retractor	Fine Science Tools	Cat#17021-13
Sterile gas compresses, 7.5cmx7.5cm	Centravet	Cat#COM421
Surgical drapes Drapvet	Centravet	Cat#CHA402
Charlotte round non-woven, non-sterile	Centravet	Cat#CHA494
Non-sterile double cotton swabs	Centravet	Cat#COT006
Shoe covers with elastic band	Centravet	Cat#COU400
High-filtration surgical mask	Centravet	Cat#MAS400
Catheter BD Insyte™ Autoguard™ 24GA 0.75IN	Becton Dickinson	#381412
PAP Pen Immunostaining marker	Dutscher	#490000
Histosette® I Tissue cassettes (45 degree angle)	Simport Scientific	#M498-2
Polysine™ Adhesion Microscope Slides, Epredia™	Avantor Sciences	#48382-117
Decloaking Chamber™ NxGen	Biocare Medical	#DC 2012

## Materials and equipment

**Table:** 

DMEM		
Reagent	Final concentration	Amount
DMEM, high glucose (4.5 g/L), glutamine-free, phenol red-free	N/A	500 mL
Fetal Bovine Serum	10%	50 mL
L-Glutamine solution	4 mM	10 mL
Sodium Pyruvate	1 mM	5 mL
Penicillin-Streptomycin	1%	5 mL

Store at 4°C under sterile conditions for up to 3 months.

**Table:** 

Analgesic solution (1.7 μL/g, 0.05 mg/kg)		
Reagent	Final concentration	Amount
Buprenorphine	0.03 mg/mL	1 mL
NaCl solution	150 mM	9 mL
Total	N/A	10 mL

Store at 4°C under sterile conditions for up to 2 weeks.

**Table:** 

95% ethanol (v/v) (Gay-lussac table)		
Reagent	Final concentration	Amount
100% EtOH	95%	100 mL
Distilled water	N/A	6.5 mL
**Total**	N/A	106.5 mL

Extemporaneous dilution.

**Table:** 

80% ethanol (v/v) (Gay-lussac table)		
Reagent	Final concentration	Amount
100% EtOH	80%	100 mL
Distilled water	N/A	28.59 mL
**Total**	N/A	128.59 mL

Extemporaneous dilution.

**Table:** 

70% ethanol (v/v) (Gay-lussac table)		
Reagent	Final concentration	Amount
100% EtOH	70%	100 mL
Distilled water	N/A	47.75 mL
**Total**	N/A	147.75 mL

Extemporaneous dilution.

**Table:** 

Tris-HCl		
Reagent	Final concentration	Amount
Trizma® hydrochloride solution 1 M	0.1 M	50 mL
Distilled water	N/A	450 mL
**Total**	N/A	500 mL

Extemporaneous dilution.

**Table:** 

Eosin		
Reagent	Final concentration	Amount
Erythrosin B	49,95 g/L	5 g
Distilled water	N/A	500 mL
Glacial acetic acid	N/A	50 μL
**Total**	N/A	500,05 mL

Store at room temperature for a maximum of 6 months.

**Table:** 

PBST		
Reagent	Final concentration	Amount
Dulbecco’s Phosphate Buffered Saline (DPBS)	N/A	100 mL
TWEEN® 20	0.1%	100 μL
**Total**	N/A	100,1 mL

Store at room temperature for a maximum of 3 months.

**Table:** 

pH 9 EDTA buffer		
Reagent	Final concentration	Amount
EDTA (ethylenediaminetetraacetic acid disodium salt dihydrate)	0,3698 g/L	0.37 g
Sigma 7-9® (Base Trizma®)	1,209 g/L	1.21 g
TWEEN® 20	0.05%	500 μL
Distilled water	N/A	1 L
Hydrochloric acid 37%	N/A	Add a volume to adjust the pH
**Total**	N/A	1.0005 L

Store at room temperature for a maximum of 3 months.

**Table:** 

GFAP antibody		
Reagent	Final concentration	Amount
GFAP antibody (dilution 1:400)	257.5 ng/mL	0.4 μL
Antibody Diluent, Dako REAL	N/A	149,6 μL
**Total (for a single histological section)**	N/A	150 μL

Extemporaneous dilution.

**Table:** 

IBA1 antibody		
Reagent	Final concentration	Amount
IBA1 antibody (dilution 1:2000)	556.5 ng/mL	0.08 μL
Antibody Diluent, Dako REAL	N/A	149,92 μL
**Total (for a single histological section)**	N/A	150 μL

Extemporaneous dilution.

**Table:** 

Goat Anti-Rabbit IgG H&L (HRP)		
Reagent	Final concentration	Amount
Goat Anti-Rabbit IgG H&L (HRP) (dilution 1:500)	4 μg/mL	0.3 μL
PBST	N/A	149,7 μL
**Total (for a single histological section)**	N/A	150 μL

Extemporaneous dilution.

## Step-by-step method details

### Orthotopic syngeneic implantation of the F98 glioma line

**Timing: 13 days**

***Note:*** The objective of the protocol below is to describe the procedure for orthotopic syngeneic implantation of the F98 glioma line by stereotaxis in the right cerebral cortex of the Fischer F344 rat.

1. F98 Cell Preparation for grafting (Timing: 1 h (3 days before grafting)).
  ***Note:*** This section describes the process of seeding F98 cells in preparation for stereotactic implantation.
  a. Remove the culture medium from the T75 cm^2^ flask by aspiration.
  b. Add 3 mL of sterile DPBS to wash the cells.
  c. Remove the DPBS by aspiration.
  d. Add 3 mL of trypsin-EDTA solution to detach the adherent cells.
  e. Incubate the flask for 5 min at 37°C in a humidified incubator (5% CO_2_).
  f. Add 4 mL of supplemented DMEM to neutralize the trypsin.
  g. Pipette gently to dissociate the cells.
  h. Centrifuge the cell suspension at 300 × *g* for 10 min at room temperature.
  i. Remove the supernatant and resuspend the cell pellet in 5 mL of fresh supplemented culture medium.
  j. Count the cells using a hemocytometer or an automated cell counter to determine the cell concentration.
  k. Seed the cells at 2 × 10^4^ cells/mL in new T75 cm^2^ flasks.
    ***Note:*** It is important to prepare one T75 cm^2^ flask per half-day of syngeneic implantation (approximately 4 rats) to ensure high cell viability at the time of implantation and promote consistent tumor development across animals.
2. Gathering the F98 cell suspension for syngeneic implantation (Timing: 1 h (immediately before grafting)).
  ***Note:*** This part describes the procedure for preparing a concentrated F98 cell suspension for stereotactic injection in rats.
  a. Remove the culture medium from the T75 cm^2^ flask by aspiration.
  b. Add 3 mL of sterile DPBS to wash the cells.
  c. Remove the DPBS by aspiration.
  d. Add 3 mL of trypsin-EDTA solution to initiate cell detachment.
  e. Incubate the flask for 5 min at 37°C in a humidified incubator (5% CO_2_).
  f. Add 4 mL of supplemented DMEM. Pipette gently to dissociate the cells.
  g. Centrifuge the resulting suspension at 300 × *g* for 10 min at room temperature.
  h. Remove the supernatant and resuspend the cell pellet in 5 mL of fresh supplemented DMEM.
  i. Count the cells using a hemocytometer or an automated counter.
  j. Calculate the total number of cells by multiplying the concentration by the resuspension volume.
  k. Centrifuge the cell suspension again at 300 × *g* for 10 min.
  l. Resuspend the final pellet in sterile HBSS (Hanks’ Balanced Salt Solution) at a concentration of 2 × 10^6^ cells/mL. This corresponds to a final injection volume of 5 μL per rat, containing 10^4^ cells.
    ***Note:*** The cell suspension must be kept on ice throughout the surgical procedure to preserve cell viability.
3. Installation of the bench and surgical equipment under aseptic conditions (Timing: 15 min).
  ***Note:*** This procedure describes the installation of equipment under sterile conditions to perform surgery.
  a. Pre-sterilize all surgical instruments using a glass bead sterilizer, then immerse them in a 0.05% chlorhexidine antiseptic bath until use.
  b. Place a sterile surgical drape on the table to organize sterile instruments, and a second drape to cover the rat once positioned in the stereotaxic frame (cut to fit the frame if necessary).
  c. Pre-cut coagulation swabs (e.g., Pangen®) into 7 × 7 mm sterile squares.
  d. Perform surgical hand antisepsis: complete two successive washes in four stages (hands → elbows → forearms → hands), maintaining forearms elevated to ensure a downward sterility gradient.
  e. Wear full sterile attire, including surgical mask, cap, protective goggles, gown, and sterile gloves changed regularly during the procedure.
    ***Note:*** The surgical field should be arranged from clean to dirty, with sterile instruments closest to the surgeon and waste containers placed peripherally.
4. Anesthesia and analgesia of the animal prior to surgery (Timing: 30 min).
  ***Note:*** The following steps describe the preparation of the animal prior to stereotactic surgery.
  a. Weigh the rat and record its body weight.
    **CRITICAL:** Ensure the weight corresponds to the expected age range (200–250 g for 7-week-old Fischer F344 males), as anesthetic doses are weight-dependent.
  b. Induce general anesthesia using isoflurane (4% at 400–500 mL/min) in an induction chamber.
  c. Maintain anesthesia at ≈ 2% via a nose cone fitted to the stereotaxic frame’s tooth bar.
    ***Note:*** Verify the absence of withdrawal reflex by lightly pinching the hind paw.
  d. Administer systemic analgesia by subcutaneous intra-scapular injection of analgesic solution (store at 4°C under sterile conditions for up to 2 weeks) (0.03 mg/mL; 0.05 mg/kg body weight; 1.7 μL/g).
    ***Note:*** Analgesia should be administered at least 15 min before incision to ensure optimal efficacy.
  e. Apply ophthalmic gel (e.g., Ocry-gel) to both eyes to prevent corneal drying.
  f. Shave and depilate the head from the neck to the eyes using clippers and depilatory cream.
  g. Place the rat in the stereotaxic frame, positioning it on a heating pad with a rectal probe lubricated with Vaseline to maintain normothermia (37.5°C) throughout surgery.
    **CRITICAL:** Hypothermia increases perioperative mortality and delays recovery.
  h. Secure the head using ear bars, ensuring proper alignment.
  i. Cover the animal’s body with sterile plastic wrap to maintain asepsis during the procedure.
  j. Administer local analgesia via subcutaneous injection of 100 μL of 2% lidocaine (e.g., Lurocaïne®, Vetoquinol, Lure, France) along the scalp in the anteroposterior axis.
  k. Clean the scalp using a sterile cotton swab soaked with chlorhexidine foam solution.
  l. Rinse the area with sterile saline and gently dry with a sterile compress.
  m. Disinfect the surgical site with 0.05% aqueous chlorhexidine.
5. Stereotactic grafting of F98 cells in the right cortex (Timing: 45 min).
  ***Note:*** This section describes the procedure for the stereotactic implantation of F98 glioma cells into the right cerebral cortex of the rat. Rats aged 7 weeks are grafted, corresponding to 200–250 g adult rats.
  a. Incise the scalp along the midline (sagittal incision), starting slightly posterior to the eyes and extending anterior to the ears, using a sterile No. 15 scalpel.
  b. Insert a retractor to expose the underlying skull.
    **CRITICAL:** Regularly rehydrate the exposed surgical field with sterile saline to prevent tissue drying.
  c. Gently remove the periosteum by scraping the skull surface with a scalpel blade or sterile cotton swab, creating a dry, clean bone surface and allowing clear visualization of cranial sutures.
  d. Attach the dental drill support onto the skull and secure it with sterile screws to stabilize the apparatus.
    **CRITICAL:** Before proceeding, verify that the skull is level by comparing the dorsoventral coordinates of the bregma and lambda landmarks (Figure 1). Slide the drill tip along the midline from bregma to lambda. The difference between the two dorsoventral readings should not exceed 0.1 mm. If misalignment is detected, adjust the height of the incisor bar to achieve proper horizontal positioning.
    **Figure 1:**
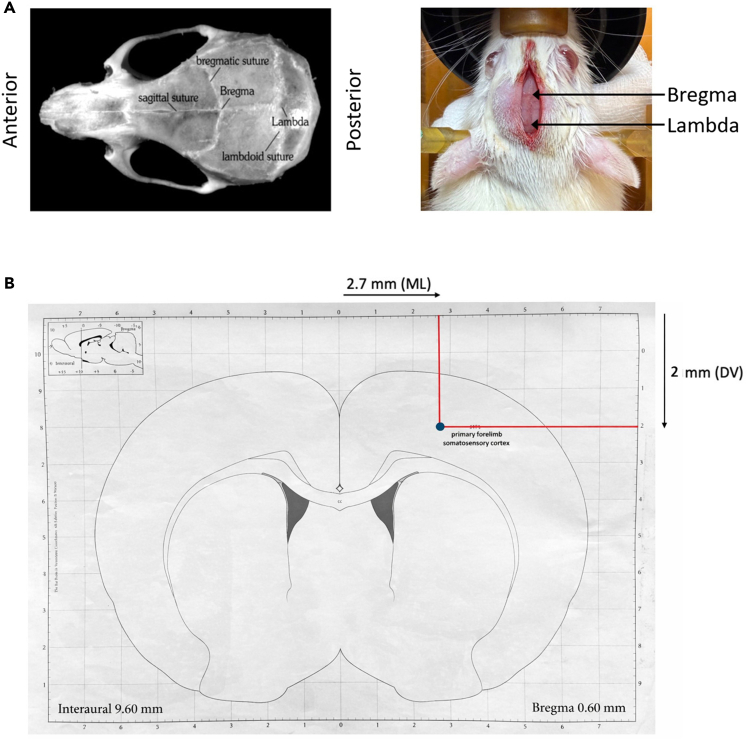
Coordinates of the tumor site in the rat right cortex (S1FL: primary somatosensory forelimb) (A) Anatomical view showing the location of bregma in rat, the reference point for stereotactic coordinates (defined as coordinate 0). (B) Coronal rat brain section extracted from the Paxinos and Watson atlas, illustrating various anatomical regions. The black arrows represent the coordinates of the F98 tumor implantation (symbolized by the blue circle) in the right primary forelimb somatosensory cortex, relative to the bregma. Implantation site (AP +0.6 mm, ML –2.7 mm, DV –2.0 mm).
  e. Position the drill tip according to the stereotactic coordinates of the implantation site: from bregma, adjust the micrometric screws to +0.6 mm along the anteroposterior (AP) axis and −2.7 mm along the mediolateral (ML) axis (Figure 1).
    **CRITICAL:** If the target point lies on the coronal suture, slightly reposition the drill posteriorly along the AP axis to minimize bleeding risk.
  f. Drill a burr hole using a 0.8 mm diameter drill bit.
  g. Use a fine sterile needle to verify penetration through the bone and gently pierce the dura mater.
    ***Note:*** In the event of bleeding, apply a sterile coagulating swab until hemostasis is achieved.
  h. Attach the nanoliter syringe pump to the stereotactic micromanipulator.
  i. Prepare the 10 μL Hamilton® syringe by rinsing thoroughly with HBSS only (never with bleach).
  j. Fill the syringe with 10 μL of F98 cells suspension, prepared at a concentration of 10^4^ cells in 5 μL of 1× HBSS (i.e., 2 × 10^6^ cells/mL).
    ***Note:*** To load the syringe, remove the plunger and needle, then fill from the top using a pipette while occluding the bottom with your thumb. Reattach the needle and reinsert the plunger once loading is complete.
  k. Verify syringe functionality by checking flow before and after injection: dispense 1 μL at the needle tip to ensure patency.
  l. Clean the external needle surface with 0.05% chlorhexidine.
  m. Mount the loaded syringe onto the injection pump, ensuring alignment with the drill hole and cortical surface.
    i. Lower the syringe needle slowly using the dorsoventral (DV) micrometer screw until the needle tip is flush with the skull surface at the center of the burr hole.
    ii. Record the DV coordinate at this point.
    iii. Use the AP micrometer screw to ensure the needle is precisely centered over the burr hole.
    iv. Advance the needle vertically into the cortex to a final DV coordinate of −2.0 mm (Figure 1B).
      **CRITICAL:** After insertion, wait 5 min to allow tissue relaxation and prevent mechanical displacement during injection.
  n. Inject the F98 cell suspension over 10 min, at a flow rate of 0.5 μL/min, for a total volume of 5 μL.
    **CRITICAL:** After injection, maintain the needle in position for an additional 10 min to minimize reflux of the cell suspension along the needle track.
  o. Withdraw the needle slowly and carefully to avoid creating a suction effect that could displace cells.
  p. Seal the burr hole using sterile bone wax to protect the injection site.
  q. Clean the skull surface with sterile saline followed by 0.05% chlorhexidine solution.
  r. Close the skin incision using sterile absorbable suture material (e.g., 4-0 Vicryl).
  s. Disinfect the wound one final time using 10% Vetedine® Solution (povidone-iodine solution, Vetoquinol, France Veto).
6. Post-surgical monitoring of the animal during the awakening period (Timing: 10 min per rat).
  ***Note:*** These stages describe the monitoring procedures for animals following surgery.
  a. Tattoo the rat for individual identification (e.g., on the tail or ear, depending on institutional guidelines).
  b. Record the animal identification number, date of surgery, and body weight on the designated monitoring sheet.
  c. Place the rat in a clean, warmed recovery cage until full emergence from anesthesia.
  d. Provide wet food (e.g., soaked chow) to facilitate mastication and hydration during the post-operative period.
  e. Monitor the animal condition daily. Humane endpoints are defined as follows:
    i. Onset of functional deficits, such as motor dysfunction detected by failure of the obstacle avoidance test (e.g., limb drag or misplacement).
    ii. Tumor diameter exceeding 5 mm on T2-weighted MRI analysis.
    iii. Body weight loss > 20% relative to pre-graft baseline.
    iv. Sustained body weight loss > 15% for two consecutive days.
    v. Deterioration of general condition, including mucosal pallor, hunched posture, immobility, vocalization, or diminished exploratory behavior.
  f. If post-operative pain is suspected, reinforce analgesia using analgesic solution at 0.05 mg/kg, subcutaneously (1.7 μL/g), administered twice on day 0 (morning and evening, 8 h apart).
7. Tumor growth monitoring with magnetic resonance imaging (MRI) (Timing: 20 min per rat).
  ***Note:*** Tumor engraftment is verified by MRI performed 8 days after implantation, and tumor growth is subsequently monitored through weekly MRI acquisitions.
  a. Induce general anesthesia using isoflurane at 4% (400–500 mL/min) in an induction chamber.
  b. Maintain anesthesia at ≈ 2% using a dedicated mask connected to the anesthesia system during image acquisition.
  c. Perform MRI acquisitions using a 3 Tesla clinical scanner (e.g., SIEMENS PRISMA) equipped with a 4 cm surface loop coil (SIEMENS, typically designed for dermatological imaging) using the parameters listed in Table 1.
    ***Note:*** Despite its clinical design, this coil provides adequate signal-to-noise ratio (SNR) and spatial resolution for small-animal brain imaging.
    ***Note:*** A fast initial acquisition is performed to localize the tumor (number of excitations (NEX) = 1). The field of view (FOV) is then adjusted to center the tumor, and a second acquisition is performed using NEX = 8 to enhance the signal-to-noise ratio.
    ***Note:*** On T2-weighted images, tumors appear as hyperintense areas in the cortical region, corresponding to increased water content and tissue disruption. When imaging is conducted on a clinical MRI system, the initial signal intensity may be insufficient to trigger acquisition. In this case, place two 2 mL Eppendorf tubes filled with saline on either side of the rat head to aid in magnetic field shimming, and maximize the shim gain in the acquisition parameters to improve signal homogeneity.

**Table 1:** Acquisition parameters for T2-weighted 2D turbo spin echo MRI

MRI modality	Matrix size/Voxel dimensions	Acquisition parameters
T2-weighted	192 × 192 × 26/0.26 × 0.26 × 0.99 mm^3^	2D turbo spin echo, TR = 2960 ms, TE = 61 ms, Flip angle = 90°, Echo train length = 6

### Surgical resection of the F98 glioma line

**Timing: 1.5 h per rat for surgery and several weeks for follow-up MRI imaging**

***Note:*** The objective of the protocol below is to describe the procedure for surgical resection of the F98 glioma in the right cerebral cortex of the Fischer F344 rat by aspiration.

8. Surgical resection of GBM by aspiration (Timing: 1.5 h per rat).
  ***Note:*** Here, we detail the surgical resection of the F98 glioma *via* aspiration to mimic clinical neurosurgical procedures, thereby providing a more clinically relevant preclinical model for the evaluation of post-surgical therapeutic strategies.
  The resection surgery is performed 10 days after F98 cell implantation, once the tumor has reached a measurable and consistent size, as confirmed by MRI.
  a. Conduct a T2-weighted MRI (as described in the section *Tumor growth monitoring with MRI Imaging*) on day 8 post-graft to verify the presence and cortical location of the tumor and to determine its volume based on maximal diameters.
  b. Perform surgery on day 10 post-implantation.
  c. Repeat all preparatory steps described in the sections *Installation of the bench and surgical equipment under aseptic conditions* and *Anesthesia and analgesia of the animal prior to surgery.*
  d. Incise the scalp along the sagittal midline, extending from just posterior to the eyes to anterior to the ears, using sterile No. 15 scalpel (Figure 2A).

**Figure 2:**
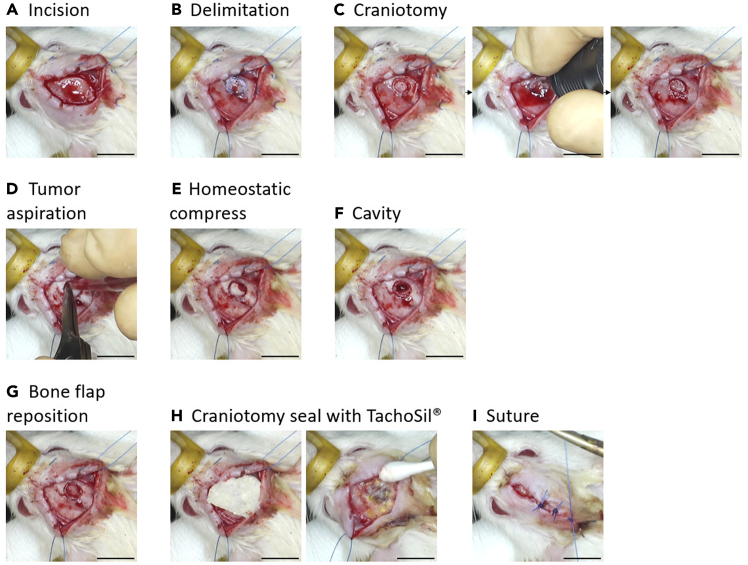
Chronology of steps for F98 glioma resection surgery in rats (A) Midline skin incision exposing the skull. (B) Delimitation of the planned bone flap using a sterile surgical marker, based on MRI measurements obtained at day 8 post-graft. (C) Drilling of the craniotomy around the marked perimeter. (D) Tumor core aspiration using a low-pressure surgical aspirator connected to a cut sterile pipette tip. (E) Application of a sterile hemostatic compress to control bleeding. (F) Visualization of the resection cavity following removal of the tumor mass. (G) Repositioning of the bone flap over the craniotomy site. (H) Sealing of the craniotomy with a saline-moistened TachoSil® patch applied with its active (yellow) side facing the surgical site. (I) Final closure of the skin incision using absorbable sutures. Scale bar = 10 mm.
  e. Insert a retractor to fully expose the skull.
    **CRITICAL:** Rehydrate the exposed surgical field regularly with sterile saline to prevent tissue desiccation.
  f. Gently remove the periosteum using a scalpel blade or sterile cotton swab to create a dry surface and allow visualization of cranial sutures.
  g. Secure the dental drill support onto the skull using sterile screws.
    **CRITICAL:** Ensure that the skull is horizontally aligned by checking DV coordinates of the bregma and lambda. The deviation should not exceed 0.1 mm.
  h. Identify the previous burr hole made during stereotactic injection 10 days earlier, which remains macroscopically visible and is typically still filled with bone wax.
  i. Using MRI measurements from day 8, outline the dimensions of the planned bone flap with a sterile surgical marker pen (Figure 2B).
    **CRITICAL:** The flap diameter should be at least equal to the tumor dimensions.
  j. Moisten the skull surface with sterile saline and drill a craniotomy centered on the previous injection site (Figure 2C).
    **CRITICAL:** Continuously hydrate the skull during drilling to prevent overheating and drill cautiously above the sagittal sinus to avoid hemorrhage from midline vessels.
  k. Remove the bone flap and immerse it in 0.05% aqueous chlorhexidine.
  l. Expose the tumor mass beneath the cortical surface.
  m. Cut the tip of a sterile pipette cone (e.g., 200 μL yellow tip) to match the tumor diameter and connect it to the tubing of a surgical aspiration system.
    ***Note:*** The surgical aspirator consists of a peristaltic pump (*e.g.* Minivac®, or equivalent low-pressure aspiration system), connected to a flexible sterile silicone tube (internal diameter ∼4 mm). Suction is applied *via* a manual footswitch to allow the operator to maintain full visual control during resection. The cut pipette tip serves as a sterile, disposable aspirating nozzle, offering good visibility and maneuverability in the surgical field.
  n. Hydrate and aspirate the tumor core, using gentle suction (Figure 2D).
  o. Control bleeding by aspirating blood and applying sterile hemostatic compresses (e.g., Pangen) (Figure 2E) to the resection cavity (Figure 2F).
  p. Rinse the bone flap, reposition it over the skull, and secure it using surgical glue along its borders (Figure 2G).
  q. Seal the craniotomy with a saline-soaked TachoSil® patch, cut to size and gently pressed in place for 5 min (Figure 2H).
    ***Note:*** TachoSil® is a sterile absorbable hemostatic patch composed of a collagen sponge coated on one side with human fibrinogen and thrombin. Upon contact with blood or physiological fluids, it activates the coagulation cascade, forming a stable fibrin clot that ensures both hemostasis and watertight sealing. In this protocol, Tachosil® is used to seal the bone flap and prevent cerebrospinal fluid leakage, which could otherwise allow extracranial migration of tumor cells and result in non-physiological subcutaneous tumor growth.
    **CRITICAL:** Ensure that the yellow (active) side of the patch is placed directly against the surgical site.
  r. Suture the skin incision with sterile absorbable thread (Figure 2I).
  s. Disinfect the wound with 10% Vetedine® solution.
  t. Provide post-operative care and monitoring as described in *Post-surgical monitoring of the animal during the awakening period.*
9. Tumor recurrence monitoring with MRI imaging (Timing: 40 min per rat).
  ***Note:*** This part presents the weekly monitoring of tumor recurrence by MRI imaging. The first acquisitions are made 12 days after surgical resection, to minimize artefacts linked to acute post-operative inflammation, which is still marked in the first few days after surgery. Acquisitions are made under the same conditions as described in the section *Tumor growth monitoring with MRI imaging*.
  Initially, a T2-weighted MRI sequence is performed with the same parameters as in section *Tumor growth monitoring with MRI imaging*.
  Then, an axial T1-weighted MRI acquisition, with injection of a gadolinium bolus, is carried out to differentiate areas of inflammation from tumor tissue in the event of recurrence. Gadolinium-enhanced imaging was specifically introduced in the post-resection setting to distinguish post-surgical inflammatory changes from true tumor recurrence, as T2-weighted sequences alone may not reliably discriminate these entities. Pre-resection imaging relied on T2-weighted sequences, which are sufficient to visualize the growing tumor mass.
  a. Induce general anesthesia using isoflurane at 4% (400–500 mL/min) in an induction chamber.
  b. Place a catheter in the animal’s tail vein for an injection of DOTAREM (native concentration 0.5 mmol/mL, 0.01 mmol/kg).
    ***Note:*** Start MRI acquisition 5 min after gadolinium injection to allow homogeneous distribution.
  c. Maintain anesthesia at ≈ 2% using a dedicated mask connected to the anesthesia system during image acquisition.
  d. Perform MRI acquisitions using a 3 Tesla clinical scanner (e.g., SIEMENS PRISMA) equipped with a 4 cm surface loop coil (SIEMENS, typically designed for dermatological imaging) using the parameters listed in Table 2.
    ***Note:*** Despite its clinical design, this coil provides adequate signal-to-noise ratio (SNR) and spatial resolution for small-animal brain imaging.
    ***Note:*** A fast initial acquisition is performed to localize the tumor (number of excitations (NEX) = 1). The field of view (FOV) is then adjusted to center the tumor, and a second acquisition is performed using NEX = 4 to enhance the signal-to-noise ratio.
    ***Note:*** On T2-weighted images, tumors appear as hyperintense areas in the cortical region, corresponding to increased water content and tissue disruption. On T1-weighted MRI with gadolinium injection, tumors show peripheral contrast enhancement, reflecting disruption of the blood-brain barrier. Hypersignal areas correspond to vascularized or active parts of the tumor, while hyposignal areas may reflect necrotic or poorly vascularized regions.

**Table 2:** Acquisition parameters for T2-and T1-weighted

MRI modality	Matrix size/Voxel dimensions	Acquisition parameters
T2-weighted	192 × 192 × 26/0.26 × 0.26 × 0.99 mm^3^	2D turbo spin echo, TR = 2960 ms, TE = 61 ms, Flip angle = 90°, Echo train length = 6
Axial T1-weighted	320 × 320 × 26/0.494 × 0.494 × 0.99 mm^3^	2D, TR = 700 ms, TE = 7.3 ms, Flip angle = 120°, Echo train length = 38

### Immunohistological characterization of F98 glioma

**Timing: 3 weeks**

***Note:*** This section describes the procedure for the histological characterization of F98 glioma in rats using hematoxylin-eosin staining and GFAP and IBA1 immunostaining to obtain an overall view of tumor structure, cellular infiltration and microglial and astrocytic response**.**

10. Brain sampling and fixation (Timing: 15 days per rat).
  ***Note:*** This segment details the collection and fixation of rat brains for immunohistological analysis.
  a. Induce general anesthesia using isoflurane at 4% (400–500 mL/min) in an induction chamber.
  b. Induce death by intraperitoneal Exagon® anesthetic overdose (initial concentration 364.6 mg/mL, 400 mg/kg and 1 μL/g).
  c. Maintain anesthesia at ≈ 2% using a dedicated mask connected to the anesthesia system until recognition of the animal’s death.
    ***Note:*** Signs of death include the absence of breathing or heartbeat, and discoloration of the mucous membranes.
  d. Proceed to the decapitation of the animal.
  e. Create a cranial flap by incising the skull edges longitudinally with scissors, before gently lifting the skull to remove the brain.
  f. Incubate the brain in 4% formalin for 2 weeks.
    ***Note:*** After 48 h, slice the brain coronally to improve fixative penetration, as F98 tumors have a necrotic core.
11. Dehydration and paraffin embedding (Timing: 1.5 days per sample).
  ***Note:*** This procedure describes the preparation of brain samples, including dehydration and paraffin embedding, with the aim of producing histological sections.
  a. Transfer the brain to an embedding cassette and immerse in 70% ethanol (extemporaneous dilution).
  b. Dehydrate the sample by placing the cassette in the automatic dehydrator Leica TP1020 for an overnight cycle. Perform the incubations described below:
    i. 45 min in 70% ethanol.
    ii. 45 min in 80% ethanol (extemporaneous dilution).
    iii. 1 h in 95% ethanol (extemporaneous dilution).
    iv. 1 h in 100% ethanol.
    v. 1 h in 100% ethanol.
    vi. 2 h in 100% ethanol.
    vii. 1 h in xylene.
    viii. 1 h in xylene.
    ix. 1.5 h in xylene.
    x. 1.5 h in paraffin.
    xi. 2 h in paraffin.
  c. Embed the sample in paraffin using the HistoCore Arcadia modular tissue embedding system.
12. Brain tissue sections (Timing: 15 min per sample).
  ***Note:*** This step provides the conditions for histological sections.
  a. Cut 5 μm sections using a microtome.
  b. Float sections on a 50°C water bath.
  c. Mount sections on Polysine™ slides.
  d. Dry overnight at 55°C.
13. Hematoxylin-eosin staining (Timing: 55 min per sample).
  ***Note:*** This chapter explains the hematoxylin-eosin staining procedure, which provides essential morphological information about tumor architecture, cell density, infiltration, and vascularization.
  a. Incubate the slide with the adhered brain tissue section according to the following sequence of steps:
    i. 5 min in toluene.
    ii. 5 min in toluene.
    iii. 5 min in 100% ethanol.
    iv. 5 min in 96% ethanol.
    v. 5 min in 70% ethanol.
    vi. 5 min in distilled water.
    vii. 8 min in Harris hematoxylin (store at room temperature for a maximum of 6 months).
      ***Note:*** Filter the colorant before use.
    viii. 4 min in distilled water.
    ix. 10 s in hydrochloric alcohol.
    x. 4 min in distilled water.
    xi. 4 min in eosin (store at room temperature for a maximum of 6 months).
      ***Note:*** Filter the colorant before use.
    xii. 4 min in distilled water.
    xiii. 3 min in 70% ethanol.
    xiv. 3 min in 96% ethanol.
    xv. 5 min in 100% ethanol.
    xvi. 5 min in toluene.
  b. Mount the slide with the coverslip using EUKITT® mounting medium.
14. Immunostaining of GFAP and IBA1 in brain tissue (Timing: 2 half-days).
  ***Note:*** This subsection describes the immunolabeling of GFAP, which allows reactive astrocytes to be visualized, highlighting changes in the glial microenvironment, and IBA1, which labels activated microglial cells, providing information on local inflammation.
  a. Deparaffinize brain tissue section by following the steps below in succession:
    i. 5 min in toluene.
    ii. 5 min in toluene.
    iii. 5 min in toluene.
    iv. 5 min in 100% ethanol.
    v. 5 min in 96% ethanol.
    vi. 5 min in 70% ethanol.
    vii. 5 min in distilled water.
  b. Unmask antigens by placing slides in a pH 9 EDTA buffer (store at room temperature for a maximum of 3 months) bath in the Biocare® autoclave (Decloaking Chamber™ NxGen), with a 40 min cycle including 5 min heating at 110°C.
    **CRITICAL:** Ensure that all slides are fully immersed and placed vertically to avoid tissue detachment during pressure cycles.
  c. Cool the bath containing the slide in a water bath for 20 min.
  d. Rinse slide with distilled water.
  e. Dry the edges of the slide and band the brain tissue section with a PAP hydrophobic pen.
  f. Permeabilize tissue with 150 μL of PBST (store at room temperature for a maximum of 3 months).
  g. Incubate for 20 min at room temperature.
  h. Eliminate PBST.
  i. Incubate 150 μL of diluted primary antibody in Dako REAL Antibody Diluent per tissue section overnight at 4°C:
    i. Dilute GFAP antibody to a final concentration of 257.5 ng/mL (equivalent to 1:400 dilution of stock solution) (extemporaneous dilution).
    ii. Dilute IBA1 antibody to a final concentration of 556.5 ng/mL (equivalent to 1:2000 dilution of stock solution) (extemporaneous dilution).
      ***Note:*** Apply enough volume to fully cover the tissue to avoid staining gradients.
  j. Perform a quick rinse with PBST.
  k. Inhibit peroxidase action by applying 150 μL of 6% H_2_O_2_.
  l. Incubate for 20 min at room temperature.
    **CRITICAL:** Do not exceed 20 min to avoid non-specific bleaching of tissue.
  m. Perform a quick rinse with PBST and then a bath of 5 min.
  n. Incubate each section with 150 μL of Goat Anti-Rabbit IgG H&L (HRP) diluted to a final concentration of 4 μg/mL (equivalent to 1:500 dilution of stock solution) in PBST (extemporaneous dilution) 1 h at room temperature.
  o. Perform a quick rinse with PBST and then a bath of 5 min.
  p. Apply 150 μL of NovaRED®.
  q. Incubate for 5 min in the dark at room temperature.
  r. Rinse the slides very quickly with distilled water.
  s. Incubate slides in a distilled water bath for 5 min.
  t. Counter-stain the brain tissue section according to the following steps:
    i. 8 min in Harris hematoxylin.
    ii. Rinse with distilled water.
    iii. 4 s in pH 8.2 0.1 M Tris-HCl (extemporaneous dilution).
    iv. Rinse with distilled water.
    v. 1 min in 100% EtOH.
    vi. 1 min in 100% EtOH.
    vii. 5 min in toluene.
  u. Mount the slide with the coverslip using EUKITT® mounting medium.
    ***Note:*** The photos were taken using the Nikon Digital Sight DS-Fi1 camera adapted to the Nikon Eclipse E600 optical microscope. Ensure consistent exposure settings across samples for quantitative comparison.

## Expected outcomes

**Figure 3:**
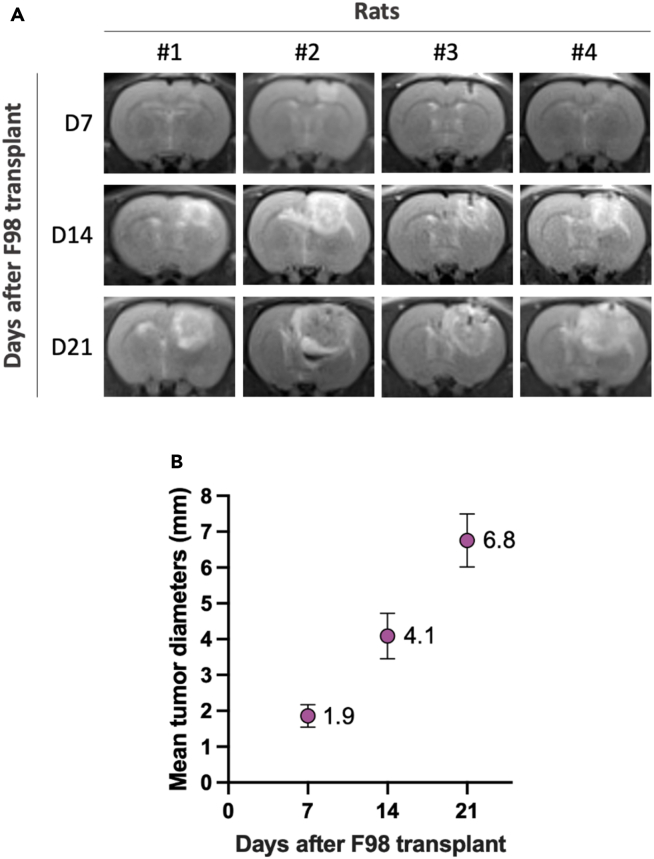
Tumor growth of F98 cells in four rats monitored by MRI (A) Representative T2-weighted coronal MRI images acquired 7 days (D7), 14 days (D14), and 21 days (D21) after stereotactic implantation of F98 cells in the right cortex (scale bar = 2 mm). Imaging was performed on a clinical 3 Tesla scanner (SIEMENS PRISMA) using a 4 cm surface loop coil, with a T2-weighted 2D turbo spin-echo sequence. Images from four individual rats (#1–#4) illustrate reproducible tumor engraftment at D7 and progressive enlargement over time. (B) Quantification of mean tumor diameters at D7 (1.9 mm), D14 (4.1 mm), and D21 (6.8 mm), measured on T2-weighted MRI. Measurements were performed on ImageJ from coronal slices centered on the tumor. Error bars represent inter-animal variability at each time point.

Following this protocol, F98 glioma cells stereotactically implanted into the right cortex of Fischer F344 rats generate reliable and MRI-visible tumors within 7–10 days (Figure 3A). On T2-weighted images, the tumor typically appears as a well-defined hyperintense cortical mass, which progressively enlarges and may show infiltration toward the underlying white matter by day 21 (D21).

Tumor growth is reproducible and follows a predictable pattern^7^: the mean tumor diameter increases from approximately 1.9 mm at day 7 (D7) to 4.1 mm at day 14 (D14) and 6.8 mm at day 21 (D21) post-implantation (Figure 3B). These values provide a reference range for researchers aiming to validate proper tumor engraftment and follow-up.

**Figure 4:**
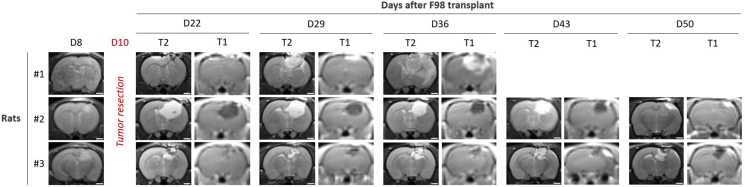
Longitudinal MRI monitoring of tumor recurrence after surgical resection of F98 glioma in three rats Engraftment was confirmed 8 days post-transplantation (D8) using T2-weighted MRI on a 3 Tesla clinical scanner (SIEMENS PRISMA) equipped with a 4 cm surface loop coil. Surgical tumor resection was performed on day 10 (D10) by aspiration. Following resection, rats were monitored weekly for potential recurrence. For each animal (#1–#3), T2-weighted and gadolinium-enhanced T1-weighted MRI acquisitions were performed at days 22 (D22), 29 (D29), 36 (D36), 43 (D43), and 50 (D50) post-transplantation (scale bar = 2 mm). DOTAREM® was injected intravenously immediately before T1 imaging to assess blood–brain barrier disruption. T2-weighted images show the post-surgical cavity and its evolution over time, while T1-weighted contrast-enhanced images highlight areas of tumor recurrence, visible as regions of peripheral or nodular enhancement. The sequential images illustrate progressive tumor regrowth in all three animals following resection.

Surgical resection by aspiration typically removes the majority of the macroscopically visible tumor mass, as assessed by early post-operative T2-weighted MRI, while preserving the surrounding parenchyma. The post-surgical cavity is clearly visible on T2-weighted images and progressively stabilizes over the following days. Tumor recurrence is commonly detectable 10–14 days after resection, presenting as a new T2-hyperintense lesion that may show contrast enhancement on T1-weighted images following gadolinium administration (Figure 4).

As in clinical neurosurgery, some variability in the extent of resection and recurrence timing is expected, owing to differences in tumor size, anatomy, and surgical exposure among animals. This variability emphasizes the importance of randomized experimental designs and monitoring tumor burden by MRI at consistent time points. Overall, this protocol yields a robust, clinically relevant model that reliably reproduces tumor growth, surgical debulking, and recurrence patterns, enabling the study of post-resection therapeutic strategies.

**Figure 5:**
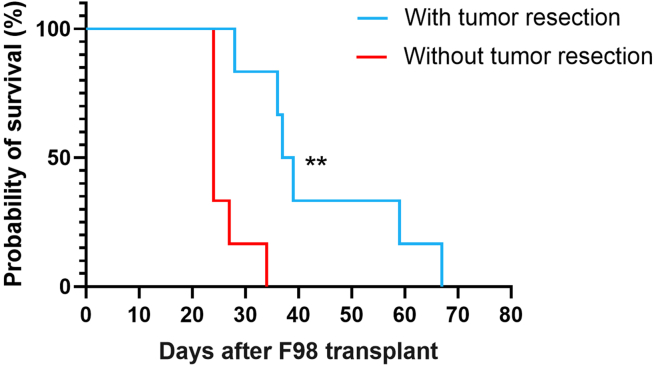
Survival curves of Fischer F344 rats bearing F98 glioma, with or without surgical resection Kaplan-Meier survival curves comparing two groups of animals (*n* = 6 per group): rats with non-resected tumors (red) and rats undergoing surgical debulking on day 10 after F98 implantation (blue). The operated group shows a clear prolongation of survival compared to the non-operated group, with median survival of 38 days versus 24 days, respectively. This model allows assessment of therapies in a clinically relevant post-resection context. ∗∗Statistical analysis was performed using the log-rank (Mantel–Cox) test (α = 0.05, *p* = 0.0015).

The development of the GBM resection model included surgical tumor removal performed 10 days after F98 cell implantation. Survival monitoring was conducted in two groups of animals (n = 6 per group): rats bearing non-resected tumors, and rats undergoing surgical resection. Animals in the resection group displayed a clear extension of survival, with median survival of 38 days compared to 24 days in the non-resected group (Figure 5). Survival curves were compared using the log-rank (Mantel–Cox) test (α = 0.05), revealing a statistically significant difference between the groups (p = 0.0015). This difference reflects the impact of tumor debulking on overall survival in this model and illustrates the usefulness of the protocol for evaluating post-surgical therapeutic strategies.

Immunohistological analysis of the F98 glioma model was carried out using GFAP and IBA1 markers. GFAP labeling identifies reactive astrocytes, providing a clear visualization of perilesional gliosis and the glial response surrounding the tumor mass. IBA1 labeling highlights activated microglial cells and infiltrating macrophages, revealing the extent and distribution of local innate immune activation within and around the tumor. Together, these markers allow detailed characterization of the glial and immune components of the tumor microenvironment and help visualize the spatial organization of cellular responses.

**Figure 6:**
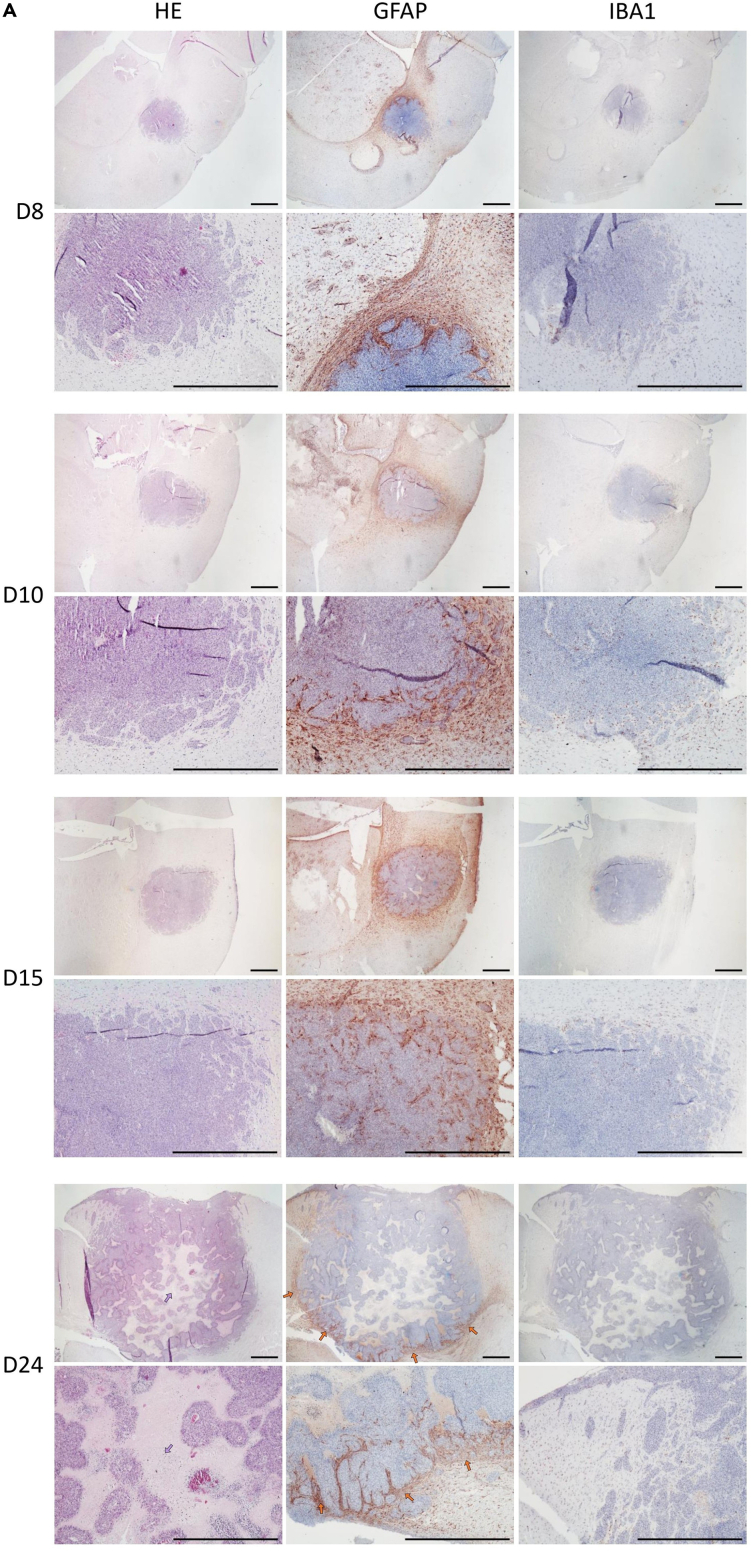
Immunohistological characterization of the primary F98 glioma model in Fischer F344 rats Representative histological and immunostaining images obtained using an optical microscope (Nikon Eclipse E600) equipped with a Nikon Digital Sight DS-Fi1 camera. Top panels: objective 1×, scale bar = 1 mm; bottom panels: objective 4×, scale bar = 1 mm. Immunohistology of primary F98 glioma tumors showing hematoxylin–eosin staining, GFAP-positive astrocytic reactivity surrounding the tumor margin, and IBA1-positive microglial/macrophagic infiltration within the tumor mass.

Hematoxylin-eosin staining revealed dense tumor cell infiltration within the brain parenchyma, associated with multiple necrotic foci (Figure 6, purple arrows).

After GFAP labeling, a high density of GFAP-positive astrocytes was observed in the periphery of the tumor, forming a gradient of reactivity, with intense labeling at the tumor margin that progressively decreased toward the surrounding healthy tissue (Figure 6, orange arrows).

**Figure 7:**
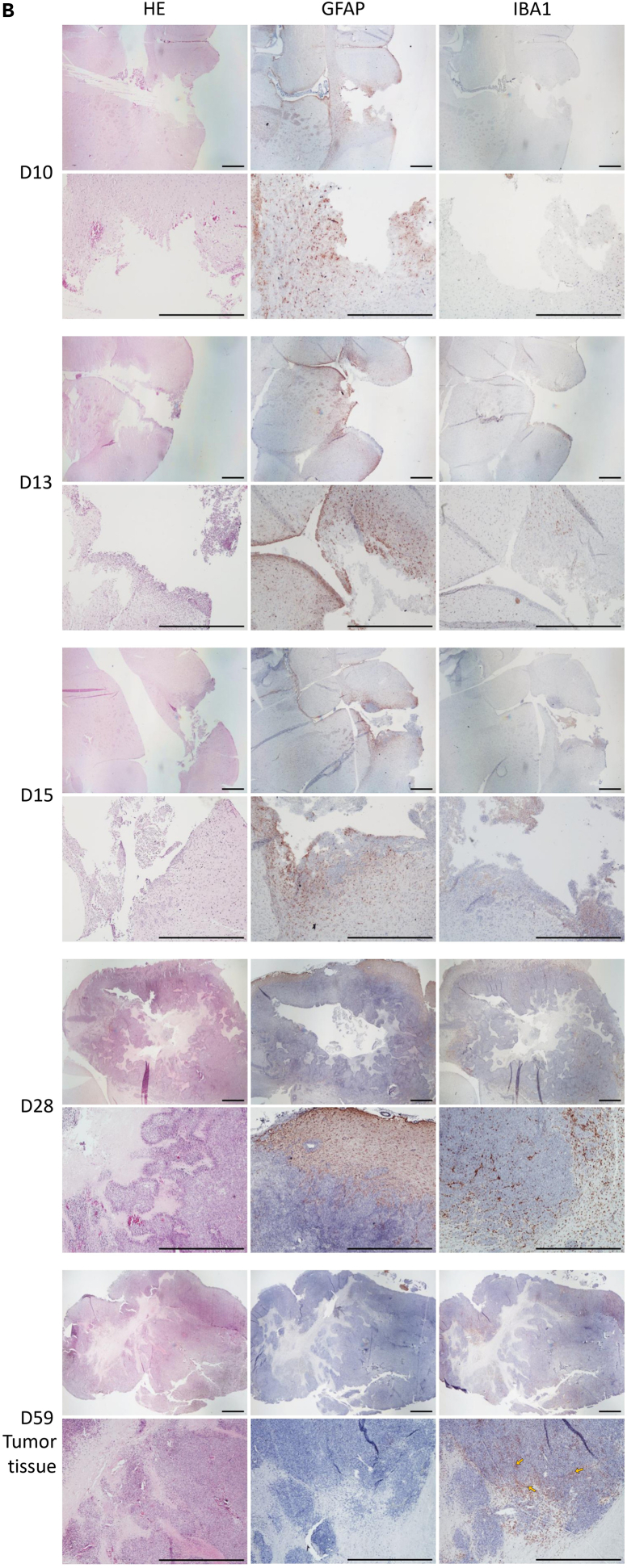
Immunohistological characterization of the recurrent F98 glioma model in Fischer F344 rats Representative histological and immunostaining images obtained using an optical microscope (Nikon Eclipse E600) equipped with a Nikon Digital Sight DS-Fi1 camera. Top panels: objective 1×, scale bar = 1 mm; bottom panels: objective 4×, scale bar = 1 mm. Immunohistology of recurrent F98 GLIOMA tumors illustrating increased necrosis, stronger GFAP labeling at the tumor border, and enhanced IBA1-positive immune cell infiltration compared to the initial lesions.

IBA1 immunostaining showed a marked presence of IBA1-positive microglial cells and macrophages within the tumor core, indicating robust immune cell infiltration in the tumor parenchyma (Figure 7, yellow arrows).

Histological features of the initial tumor were similar to those observed in recurrent tumors, although recurrence was associated with increased cellular infiltration, more extensive necrotic areas, and an elevated density of both GFAP- and IBA1-positive cells over time (Figure 7).

## Limitations

This protocol involves technically demanding procedures, including stereotactic cell implantation and microsurgical tumor resection, both of which may introduce inter-operator variability. MRI follow-up requires access to high-resolution imaging equipment and dedicated small-animal anesthesia systems, which may limit reproducibility across facilities. The F98 glioma model is syngeneic, highly infiltrative, and resistant to radio- and chemotherapy, but it does not reproduce the full spectrum of molecular alterations observed in human GBM, such as EGFR amplification. Therefore, the findings obtained with this protocol reflect the biological characteristics of this specific model. In addition, other GBM cell lines may exhibit distinct growth kinetics, infiltrative properties, and therapeutic sensitivities. Consequently, the optimal timing and extent of surgical resection may differ depending on the tumor model used.

## Troubleshooting

### Problem 1

No tumor visible on MRI at D7 post-graft (step 7 and 9).

### Potential solution

Confirm cell viability at the time of injection. Ensure accurate stereotactic positioning and prevent reflux by waiting 5 min before, and 10 min after injection before withdrawing the needle.

### Problem 2

Excessive bleeding during cranial drilling (step 5 and 8).

### Potential solution

Avoid drilling close to the sagittal sinus. Keep the skull surface hydrated with sterile saline, reduce drill speed, and apply minimal pressure.

### Problem 3

Post-operative neurological deficit (step 8).

### Potential solution

Check that resection depth has not exceeded the tumor boundaries. Ensure correct placement of the TachoSil® patch to protect cortical tissue. Monitor recovery according to institutional animal-welfare guidelines.

### Problem 4

Poor MRI signal or artifacts (step 7 and 9).

### Potential solution

Place two 2 mL saline-filled Eppendorf tubes on either side of the rat’s head to improve magnetic field shimming. Increase the number of excitations (NEX) to enhance signal-to-noise ratio.

### Problem 5

No tumor recurrence following resection (step 8 and 9).

### Potential solution

Confirm that the initial tumor was present and well-defined on pre-operative MRI. Excessive aspiration may remove infiltrative tumor margins; ensure resection follows the planned surgical margins based on MRI guidance.

## Resource availability

### Lead contact

Requests for further information and resources should be directed to and will be fulfilled by the lead contact, Muriel Barberi-Heyob (muriel-barberi@univ-lorraine.fr).

### Technical contact

Technical questions on executing this protocol should be directed to and will be answered by the technical contacts, Perrine Schneller (perrine.schneller@univ-lorraine.fr) and Julien Pierson (julien.pierson@univ-lorraine.fr).

### Materials availability

This study did not generate new unique reagents.

### Data and code availability

This study did not generate new unique codes.

## Acknowledgments

We thank the Animalerie du Campus Biologie Santé (ACBS, Nancy) for animal care and the 3 T IRM imaging platform for access and technical support. This work was supported by the 10.13039/501100001665French National Research Agency (10.13039/501100001665ANR) under the project IRHydroBRAIN (grant no. ANR-24-CE19-6774-01) and by the HydroRI project funded by the Cancéropôle Est.

## Author contributions

P.S., J.P., J.D., S.P., and M.B.-H. designed the protocol, supervised the animal work, and wrote and revised the manuscript. P.S., J.P., and F.R. performed surgeries and optimized the stereotactic procedure. P.S., J.D., J.P., and M.D. conducted MRI acquisitions and analysis. P.S., J.P., and M.B.-H. contributed to data interpretation and histological validation. All authors read and approved the final manuscript.

## Declaration of interests

The authors declare no competing interests.
